# Development of a sensitive PCR-dot blot assay to supplement serological tests for diagnosing Lyme disease

**DOI:** 10.1007/s10096-017-3162-x

**Published:** 2017-12-27

**Authors:** J. S. Shah, I. D’ Cruz, S. Ward, N. S. Harris, R. Ramasamy

**Affiliations:** IGeneX Inc., 795 San Antonio Road, Palo Alto, CA 94303 USA

## Abstract

Laboratory diagnosis of Lyme disease is difficult and presently dependent on detecting *Borrelia burgdorferi*-specific antibodies in patient serum with the disadvantage that the immune response to *B. burgdorferi* can be weak or variable, or alternatively, the slow and inefficient culture confirmation of *B. burgdorferi*. PCR tests have previously shown poor sensitivity and are not routinely used for diagnosis. We developed a sensitive and specific Lyme Multiplex PCR-dot blot assay (LM-PCR assay) applicable to blood and urine samples to supplement western blot (WB) serological tests for detecting *B. burgdorferi* infection. The LM-PCR assay utilizes specific DNA hybridization to purify *B. burgdorferi* DNA followed by PCR amplification of *flagellin* and *OspA* gene fragments and their detection by southern dot blots. Results of the assay on 107 and 402 clinical samples from patients with suspected Lyme disease from Houston, Texas or received at the IGeneX laboratory in Palo Alto, California, respectively, were analyzed together with WB findings. The LM-PCR assay was highly specific for *B. burgdorferi.* In the Texas samples, 23 (21.5%) patients antibody-negative in WB assays by current US Centers for Disease Control (CDC) recommended criteria were positive by LM-PCR performed on urine, serum or whole blood samples. With IGeneX samples, of the 402 LM-PCR positive blood samples, only 70 met the CDC criteria for positive WBs, while 236 met IGeneX criteria for positive WB. Use of the LM-PCR assay and optimization of current CDC serological criteria can improve the diagnosis of Lyme disease.

## Introduction

Lyme disease caused by the spirochete bacterium *Borrelia burgdorferi* (BB) is endemic in many parts of the world and the most prevalent tick-borne disease in Europe and the United States [1–3]. A ‘bulls-eye’ rash or erythema migrans (EM) is generally the best indicator of an early acute infection [4, 5]. However EM is observed in only 60–80% of BB infections [4, 5]. Other common symptoms of acute BB infections are arthralgia, fatigue, fever, headache, mild stiff neck and myalgia [4, 5]. Cardiac, dermatologic, musculoskeletal and neurological manifestations may be evident in advanced stages of Lyme disease [4, 5]. Patients with chronic fatigue syndrome, fibromyalgia, immune disorders, multiple sclerosis or infections with other microorganisms may present with Lyme disease-like symptoms, adding to the importance of accurately diagnosing Lyme disease in the laboratory.

The laboratory diagnosis of Lyme disease is also difficult and the appropriate procedure is a matter of current debate. A two-tiered serological test based on an enzyme-linked immunosorbent assay (ELISA) or an indirect immunofluorescence assay (IFA) followed by a western blot of samples that test positive or equivocal by ELISA or IFA, or alternatively the culture confirmation of BB, are presently recommended by the US Centers for Disease Control (CDC) for laboratory diagnosis [5, 6]. ELISA tests however have limited specificity and sensitivity [7] and about 20–30% of patients do not make detectable antibodies to BB [8]. Diagnosis of Lyme disease by culturing BB is a tedious and inefficient procedure [9, 10]. Studies on early Lyme disease reported PCR assays on skin biopsies to be a sensitive diagnostic method [9]. PCR assays on synovial and cerebrospinal fluids have proved to be useful diagnostic tools for Lyme arthritis and neuroborreliosis, respectively [10–13]. Urine and blood are PCR tested in Lyme disease less frequently [12] because of poor sensitivity partly due to the presence of PCR inhibitors, and a lack of reproducibility [14, 15]. A nested PCR assay for urine samples was found to be sensitive [16] unlike a quantitative one-step real-time PCR [17] with the results suggesting that sample handling preparation, and the extraction of DNA were limiting factors for urine [16, 17].

The use of specific oligomer probes to select, purify and concentrate target DNA from the clinical samples, and at the same time remove PCR inhibitors, has been reported to improve the sensitivity of PCR assays for some other infections [18–21]. A hybrid-select multiplex dot blot PCR assay for Lyme disease, termed the Lyme multiplex PCR-dot blot assay (LM-PCR assay) was therefore developed in an attempt to improve the sensitivity of the PCR-based detection of BB in blood and urine samples. The LM-PCR assay targets the BB genome-encoded *flagellin* gene and a BB plasmid-encoded *OspA* gene for detection in a final southern dot blot of the corresponding PCR amplified products. We describe here the development of the LM-PCR assay and analyze its laboratory diagnostic performance in relation to western blots in clinical samples tested for Lyme disease.

## Materials and methods

### Origins of analyzed clinical samples

#### Texas samples

Results obtained from coded samples of 107 individuals with Lyme-like symptoms on presentation, not vaccinated against Lyme disease, living in Lyme disease-endemic areas of Southeastern Texas, and identified by a physician in Houston, TX were used for the first set of analyses. Five individuals from this cohort were aged between 0 and 5 years, 26 were between 5 and 21 years, and 76 between 21 and 88 years. None of the selected individuals remembered having an EM rash, had previously undergone laboratory tests for Lyme disease, or been treated for Lyme disease when the clinical samples were collected. Serum, EDTA-treated whole blood, and urine preserved with 0.1% Boric acid (BD Diagnostics Systems, Bethesda, MD) collected from this study population were sent from Houston by courier mail to the IGeneX laboratory in Palo Alto, CA where all the samples were tested by both LM-PCR and western blot assays.

#### IGeneX samples

A subsequent study analyzed the results of all LM-PCR positive coded samples of whole blood and serum from 5,964 patients suspected by physicians to have Lyme disease and received at the IGeneX laboratory over a two-year period for testing by both LM-PCR and western blots. Of the 5,964 samples, only 402 patient samples were positive for BB in the LM-PCR assay and these were then analyzed in relation to their western blot findings.

### Lyme western blot assays

Five mm-wide western blot strips were prepared in the IGeneX laboratory with pooled 1:1 mix of sonicated cell lysates of BB isolates B31 (ATCC 35210) and 297 (originating from Dr. Denne Thomas, University of Texas, San Antonio, TX) and validated with positive and negative control human sera and rabbit antibodies to BB as previously described [22]. Each patient’s serum was then tested on the strips by Lyme western blot IgG and IgM assays essentially by previously described methods [22]. Briefly, an aliquot of 10 μl of the patient’s serum sample diluted in PBS to 1 ml was separately incubated with test strips for subsequent tests for IgG and IgM antibodies, for 1 h at room temperature. For IgG western blots, washed strips probed with human sera were incubated with alkaline phosphatase-conjugated goat anti-human IgG (Sera Care, Gaithersburg, MD) for 1 h at room temperature. For IgM western blots, washed strips probed with human sera were incubated with alkaline phosphatase-conjugated goat anti-human IgM (Sera Care, Gaithersburg, MD) for 1 h at room temperature. Excess conjugate was aspirated and the strips were washed three times with the wash buffer at room temperature. One ml 5-bromo-4-chloro-3-indolylphosphate/nitroblue tetrazolium was then added to the strips and incubated for 10 min at room temperature for color development. Tests with patient sera were considered acceptable if the positive control human serum showed all the antigen bands and the negative control human serum did not show any of those bands in parallel test strips. Test western blots were scored using a calibration control with alkaline phosphatase-conjugated rabbit anti-BB antibody to the 39 and 93 kDa antigens (Strategic Biosciences, Stow, MA) so that bands with lower intensity than those of this calibration control in test blots were considered to be negative [22]. The western blots were read by CDC criteria [5, 6] and in-house criteria developed at IGeneX that were based on previous observations [8, 22, 23]. The IgG western blot was considered positive according to the CDC criteria if at least five of the ten BB-specific bands of 18, 23-25, 28, 30, 39, 41, 45, 58, 66 and 93 kDa were present [5]. The IgM western blot was considered positive according to the CDC criteria if at least two of the three BB-specific bands of 23-25, 39, and 41 kDa were present [5]. By IGeneX criteria, IgG western blots were considered positive if two or more of the six BB-specific bands of 23-25, 31, 34, 39, 41, 93 kDa were present, while IgM western blots were considered positive if two or more of the five BB-specific bands of 23-25, 31, 34, 39, 41 were present [22].

### Specificity controls with other pathogens for the LM-PCR assay

#### Control bacterial and parasite cells

*Ehrlichia chaffeensis* (ATCC 1455VR/Lot 1 W), *Bartonella henselae* (ATCC 49882/1436056), *Bartonella elizabethae* (ATCC 499271/1192868), *Bartonella quintana* (ATCC 51694/026125), *Bartonella clarridgeiae* (ATCC 51734/1284170), *Staphylococcus aureus* (ATCC 25923/1958041), *Escherichia coli* (ATCC 25922/1980205), *Pseudomonas aeruginosa* (ATCC 27853/1906403), *Haemophilus influenzae* (ATCC 19418/1574755), *Staphylococcus bovis* (ATCC 1165750), *Listeria monocytogenes* (ATCC 191115/20122438), *Plasmodium falciparum* (ATCC 30932/SF 2195) and *Babesia microti* infected hamster cells (AS 101901) as frozen cells were purchased from American Type Culture Collection (ATCC). The frozen cells were washed twice in PBS, followed by proteinase-K treatment at 60 °C for 1 h. Proteinase K was then inactivated by heating at 100 °C for 10 min. The processed cell lysate was diluted serially in TE-glycogen (10 mM Tris, 1 mM EDTA and 1% glycogen, pH 8.0).

#### Other controls

*Trypanosoma cruzi* and *Leishmania donovani* cell lysates were provided by Dr. George Steward, Southern Florida University, FL. *Trypanosoma brucei*, *Trypanosoma gambiense*, *Toxoplasma gondii* and *Cryptococcus neoformans* DNA were provided by Dr. John Bootheroyd, Stanford University, CA. *Plasmodium vivax* and *Plasmodium malariae* DNA were provided by the Wellcome Trust Laboratory, Nairobi, Kenya.

### Processing of clinical specimens for the hybrid-select procedure

#### Serum and EDTA-treated whole blood samples

An aliquot of 200 μl of either the serum or blood sample was mixed with 200 μl of Sample Processing Buffer (SPB - 100 mM Tris-HCl, pH 7.4, 40 mM EDTA, 5 M guanidine thiocyanate and 1.0% sarkosyl).

#### Urine samples

One ml of urine sample was centrifuged at 13,000 g for 10 min at room temperature and 900 μl of the supernatant was removed. The remaining concentrated 100 μl of urine was mixed with 100 μl of SPB.

### Hybrid-select procedure for purifying BB DNA

A set of three capture probes labeled with biotin at the 5′ terminus synthesized by Operon Technologies/Qiagen, Alameda, CA, were used for hybrid selection. These were Probe A: 5’-Biotin- GCC -TTA –ATA-GCA -TGT -AAG –CAA- AAT- GTT- AGC- AGC-CTT GAT -3′; Probe B: 5’-Biotin-TCC-ATC-GCT-TTT-AAT-TCC-TGT-GTA-TTC-AAG-TCT-GGT-TCC-3′, and Probe C: 5’-Biotin- ATC TGT AAT TGC AGA AAC ACC TTT TGA AT -3′. Probe A and Probe B were designed to selectively bind *OspA* and Probe C to *flagellin* gene sequences respectively that are conserved in different BB strains.

Fifty μl of a 1 μg/ml solution of each of the capture probes were added to each of the processed sample in a tube and mixed. The tubes were incubated at 85 °C for 10 min to denature the DNA, followed by incubation at 37 °C for 3 h for hybridization of the biotin labeled probes to BB DNA. The hybrids were captured on the Magnesphere® paramagnetic particles derivatized with streptavidin (Promega, Madison, WI). The target DNA-probe hybrid bound to the beads was washed three times with wash buffer (0.1xSSC, 0.1% Sarkosyl, 0.1% BSA, pH 6.8) for 5 min each at 37 °C to remove excess probe, PCR inhibitors and cell debris. The target DNA-probe hybrid was then dissociated from the beads by adding 100 μl of 10 mM Tris buffer and incubating at 65 °C for 10 min. The beads were then separated from the DNA by centrifugation.

### PCR amplification

Purified DNA was amplified using two sets of primers, an *OspA* primer set described by Mouristen et al. (primer 1: 5′-AAG-CAA-AAT-GTT-AGC-AGC-CTT-GA-3′ and primer 2: 5’-CTT-TGT-TTT-TTT-CTT-TGC-TTA-CAA-GAA-C-3′) [24], and a *flagellin* gene-specific primer set described by Rosa et al. (primer 3: 5′-GAA-TTA-AAT-TTT-GGC-TTG-TCA-GGA-GCC-TAT-GG-3′ and primer 4: 5’-GCT-TTT-TTG-TTA-GGA-TCT-GAG-GGT-GTT-TCT-TT-3′) [25]. The primers were synthesized by Operon Technologies/Qiagen, Alameda, CA. Two PCR reactions were performed on each sample. One PCR was performed at the annealing temperature of 62.7 °C determined to be optimal for the *flagellin* gene. A second PCR was performed at an annealing temperature of 59 °C determined to be optimal for the plasmid-encoded *OspA* gene. For each PCR, 9 μl of the DNA prepared by the hybrid select procedure were combined with 21 μl of the PCR reaction mixture (10 mM Tris-HCl pH 8.3, 50 mM KCL, 5 mM MgCl2, 0.001% gelatin, 1% glycerol, 0.5 μg of each of the primers, 0.1 mM dNTPs, and 0.25 U Amplitaq Gold from Applied Biosystems, Foster City, CA). PCRs were performed following an initial denaturation at 95 °C for 10 min in an Eppendorf Thermocycler 7000 for 50 cycles using a denaturation at 95 °C for 1 min, annealing at 62.7 °C or 59 °C for 1 min and extension at 72 °C for 1 min. This was followed by a final 10 min extension at 72 °C.

### Dot-blot analysis

The amplified PCR products were detected in a final dot-blot assay using two BB specific 5′ digoxigenin (DIG) labeled probes using the Roche hybridization kit (Roche Molecular Diagnostics, Pleasanton, CA, USA). Probe 1 (5’-TTC TGC AAT TTT AGC ATC TTT TGG AGC TAA ATA TAA GCT TGG AT-3′) targets the genomic *flagellin* gene and Probe 2 (5′-GAA AAA CAG CGT TTC AGT AGA TTT GCC TGG TGA AAT GAA-3′) targets the plasmid-encoded *OspA* gene.

Briefly the PCR products were denatured by the addition of 20 μl of 0.4 M NaOH to 20 μl of PCR products. A 10 μl aliquot of the denatured PCR product was transferred to nitrocellulose membrane using a dot-blot apparatus (Biorad, Hercules, CA), rinsed in 6× SSC buffer and air-dried. The air-dried membranes were placed in a vacuum oven at 80 °C for 1 h to fix DNA onto the membrane. The DNA bound on membranes was hybridized to a mixture of the two *BB*-specific DIG – labeled oligonucleotide probes targeting the two different PCR products in 50% formamide hybridization buffer overnight, at 42 °C, according to the kit manufacturer’s instructions (Roche Molecular Diagnostics, Pleasanton, CA, USA), washed and then treated with an anti-DIG alkaline phosphatase conjugate. A subsequent enzyme-catalyzed color reaction with 5-bromo-4-chloro-3-indolyl phosphate and nitroblue tetrazolium salt produced an insoluble blue precipitate on the membrane in the PCR products of all the positive controls and patient samples that had a *BB*-specific PCR product. A test run for a clinical sample was only considered acceptable if the positive control was positive and the negative control was negative on the same blot.

### Agarose gel electrophoresis of PCR amplicons

PCR amplified products from a random selection of patient samples that were positive by LM-PCR were analyzed by gel electrophoresis on a 3% agarose gels in Tris-borate, EDTA pH 8.1 (Biorad, Milwaukee, WI), at a constant voltage of 140v for 45-60 min. A positive control and a negative control were included in every run. Gels were stained with ethidium bromide, and DNA was visualized by UV transillumination. A sample was considered positive if there was a PCR product present at 320 bp corresponding to the amplicon from the *flagellin* gene and 93 bp corresponding to the amplicon from the *OspA* gene in the agarose gel in the two PCRs carried out with annealing temperatures of 62.7 °C and 59 °C respectively.

### Limit of detection of BB cells in the LM-PCR assay

Aliquots of 5 μl of the serially diluted proteinase K-treated BB B31 cell lysate at concentrations of 10^6^ to 10^−3^ cell equivalents per ml was purified by the hybrid-select protocol, PCR-amplified and then tested by dot blotting in the LM-PCR assay.

### Comparison of the limits of detection of BB DNA isolated by the IGeneX hybrid-select and Gentra column protocols from clinical samples in the LM-PCR assay

Cell lysates from 1, 10^1^, 10^2^, 10^3^ and 10^4^ BB cells were used to spike in triplicate whole blood (0.2 ml), serum (0.2 ml) and urine (1 ml) collected from three uninfected control persons. DNA from the spiked samples were either purified on Gentra DNA-purification columns (Gentra Systems, Minneapolis MN) according to the manufacturer’s instructions or by the IGeneX hybrid-select protocol. The purified DNA from each sample was PCR amplified and the BB-specific amplified products were detected in the LM-PCR assay.

### Additional validation of the LM-PCR assay

The LM-PCR assay was additionally validated by DNA sequencing the PCR-amplified products for *OspA* and *flagellin* gene sequences from 20 randomly selected (approximately 10% of total sample) LM-PCR assay-positive clinical samples that had been received at IGeneX prior to commencing tests on the Texas and IGeneX samples.

The possible presence of PCR inhibitors in DNA purified from clinical samples was tested on a set of 282 patient samples (94 each of whole blood, serum and urine) that were negative by LM-PCR, and received at IGeneX prior to commencing studies on the Texas and IGeneX samples. These were spiked with BB plasmid DNA carrying *OspA* at ten-fold higher concentration than the limit of detection. DNA from the samples were then purified by hybrid-select, PCR-amplified with *OspA*-specific primers and the *OspA* amplicon detected by dot blotting as for LM-PCR assays.

### Statistical analysis

Differences in proportions of test sera that reacted in western blots were determined by the chi-square test with Yate’s correction for sample sizes ≤5.

## Results

### Specificity of the LM-PCR assay for *Borrelia burgdorferi*

DNA from *Babesia microti, Bartonella clarridgeiae, Bartonella elizabethae, Bartonella henselae, Bartonella quintana, Cryptococcus neoformans, Ehrlichia chaffeensis, Anaplasma phagocytophila, Escherichia coli, Haemophilus influenza, Leishmania donovani, Listeria monocytogenes, Plasmodium falciparum, Plasmodium malariae, Plasmodium vivax, Pseudomonas aeruginosa, Staphylococcus aureus, Staphylococcus bovis, Toxoplasma gondii, Trypanosoma gambiense, Trypanosoma brucei,* and *Trypanosoma cruzi* were all negative when tested by the LM-PCR assay. In addition, a set of 100 urine samples, ten whole blood samples and ten serum samples collected from known uninfected controls between the ages of 1 and 60 years were tested and found to be negative in the LM-PCR assay. Thus, the specificity of the LM-PCR assay with the tested controls was 100%.

### Limit of detection of BB cells in the LM-PCR assay

**Fig. 1:**
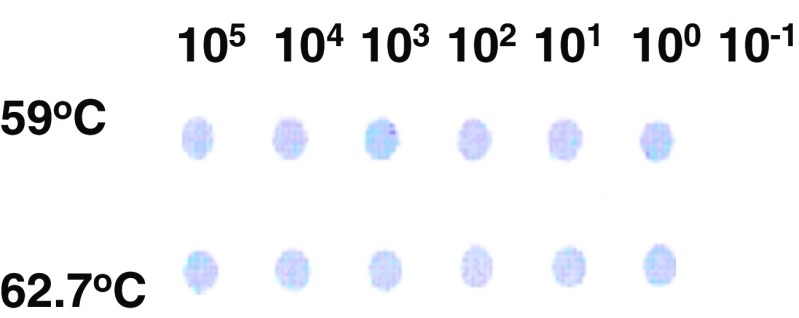
Limit of detection of *B*. *burgdorferi* by dot blots in the LM-PCR assay. Proteinase K treated cell lysates derived from different numbers of *B. burgdorferi* from culture were purified by hybrid-selection and PCR amplified using an annealing temperature of 59 °C (*top row*) and 62.7 °C (*bottom row*) and then dot blotted. Results with *B. burgdorferi* cell lysates from the equivalent of 10^5^, 10^4^, 10^3^, 10^2^, 10^1^, 10^0^, and 10^−1^ cells are shown in each of the correspondingly labeled columns

**Fig. 2:**
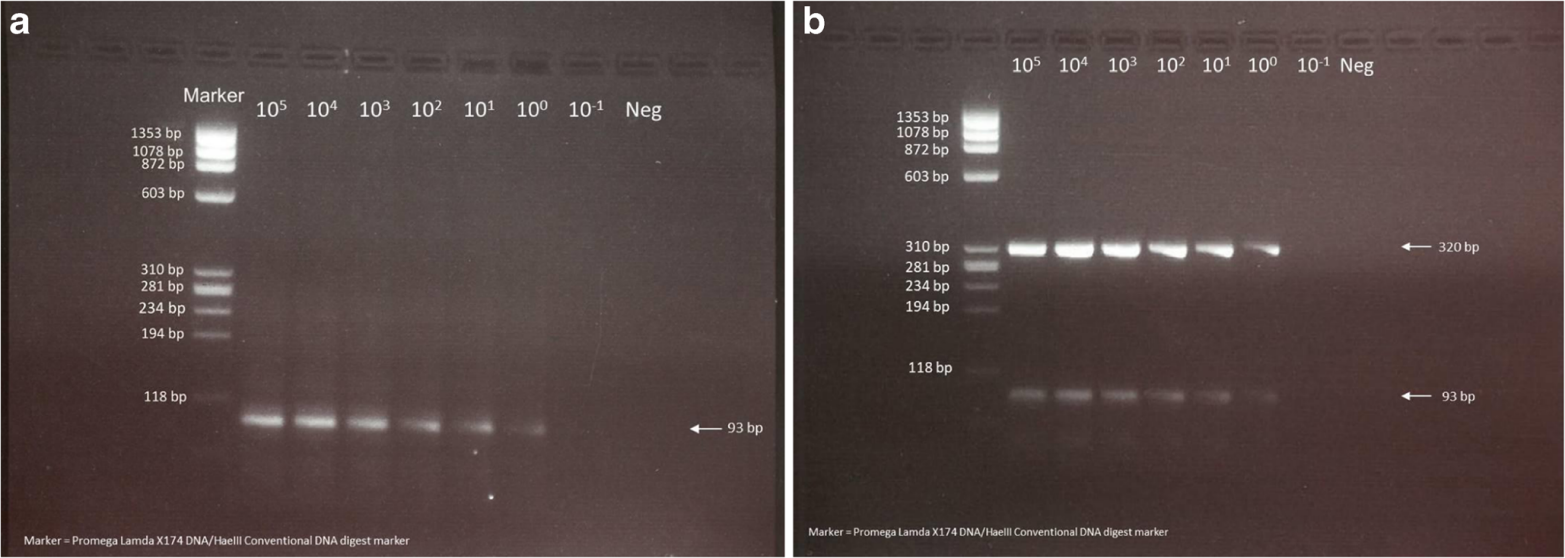
Limit of detection by agarose gel electrophoresis of the PCR amplified products from *B*. *burgdorferi* produced in the LM-PCR assay. Proteinase K treated cell lysates of cultured *B. burgdorferi* were purified by hybrid-selection and PCR amplified at annealing temperatures of (A) 59 °C for the 93 bp fragment of the *OspA* gene, and (B) 62.7 °C for the 320 bp fragment of the *flagellin* gene. The PCR amplicons were then analyzed on 3% agarose gels. *Marker* Lambda X174 *Hae* III digest molecular size marker. The equivalent *B. burgdorferi* cell numbers ranging from 10^5^ to 10^−1^ used to derive samples electrophoresed in different lanes are shown at the top of each lane. *Neg* negative control without *B. burgdorferi* cell lysate

The dot blot results in the LM-PCR assay obtained with PCR amplified products from 10^5^ to 10^−1^ BB cells are shown in Fig. 1. The observed limit of detection for both genomic *flagellin* and plasmid-encoded *OspA* genes was 1 BB cell. The same limit of detection of 1 BB cell, was seen when the PCR products used in the LM-PCR assay were analyzed on agarose gels with ethidium bromide staining (Fig. 2). It was observed that the 93 bp amplicon from the *OspA* gene, for which the optimal annealing temperature was 59 °C, was also amplified more weakly in the PCR where the annealing temperature was 62.7 °C (Fig. 2).

### Limit of detection of BB DNA isolated by the IGeneX hybrid-select and Gentra column protocols in the LM-PCR assay

The results (Table 1) showed that the IGeneX hybrid selection procedure for isolating BB DNA from clinical samples was at least 10^2^ fold more efficient for urine, about ten fold more efficient for serum and about as efficient for whole blood, when compared with the widely-used Gentra column method for purifying DNA.

**Table 1:** Comparison of the limits of detection of *Borrelia burgdorferi* DNA purified by the Gentra column binding and IGeneX hybrid-selection methods in the LM-PCR assay

Sample	LM-PCR assay limit of detection (*Borrelia burgdorferi* cells per sample)	
	Gentra	IGeneX
Urine (1 ml)	10^3^ –10^4^	1–10
Serum (0.2 ml)	10^1^–10^2^	1–10
Whole blood (0.2 ml)	1–10	1–10

### Additional validation of the LM-PCR assay

All PCR-amplified DNA from randomly chosen LM-PCR assay-positive clinical samples that were sequenced were confirmed to be from BB genes indicating the clinical specificity of the LM-PCR assay. Furthermore, inhibition of PCR amplification was not detectable when 94 samples each of whole blood, serum and urine were spiked with BB plasmid DNA before PCR amplifying the *OspA* fragment and detecting the amplified product by dot blot in the LM-PCR assay.

### Results of LM-PCR and western blot assays on clinical samples from Texas

All serum samples were tested for Lyme disease by IgG and IgM western blots and read by both CDC and IGeneX criteria. EDTA whole blood and/or serum samples from all patients and urine samples from 105/107 patients were tested by the LM PCR assay. The findings with the 107 clinical samples from Texas are summarized in Table 2.

**Table 2:** Analysis of LM-PCR assay and western blot results with clinical samples from Texas

Test	Western blot results by IGeneX criteria (CDC criteria)				
	Only IgM antibody positive	Only IgG antibody positive	Both IgG and IgM antibody positive	IgG and IgM antibody negative	Total number of patients
Total LM-PCR –ve	17 (17)	6 (6)	8 (8)	41(41)	72 (72)
LM-PCR + ve Urine only	7 (3)	6 (3)	0 (0)	8 (15)	21(21)
LM-PCR + ve Blood only	5 (3)	3 (2)	0 (0)	1(4)	9 (9)
LM-PCR + ve Blood and Urine	1 (1)	0 (0)	1 (0)	3 (4)	5 (5)
Total LM-PCR + ve	13 (7)	9 (5)	1(0)	12 (23)	35 (35)
Total for each column	30 (24)	15 (11)	9 (8)	53 (64)	107

The numbers of samples deemed to be positive using the IGeneX criteria are shown with corresponding numbers positive using the CDC criteria as shown in *parenthesis* in each case

Forty-three samples from Texas were positive by the CDC and 54 by the IGeneX western blot criteria for IgG and/or IgM anti-BB antibodies. This represents 40.2% and 50.5%, respectively, of the 107 patients tested. Of the 43 samples that had serologically-detectable antibodies by the CDC criteria, 32 (74.4%) had IgM and 19 (44.2%) had IgG antibodies. Of the 54 samples that had serologically detectable antibodies by the IGeneX criteria, 39 (72.2%) had IgM and 24 (44.4%) had IgG antibodies. The 19 and 24 patients with IgG antibodies to BB by the CDC and IGeneX criteria, represented 17.8% and 22.4%, respectively, of the entire cohort from Texas.

The 35 patients tested positive with either blood or urine samples in the LM-PCR assays represented 32.7% of the sample cohort. Twenty six out of the 35 (74.3%) LM-PCR positive samples tested positive in urine. Infection with BB could not be detected by either western blotting or LM-PCR in 41 individuals representing 38.3% of the cohort.

In patients positive in the LM-PCR assay, the use of the IGeneX criteria yielded a significantly higher proportion of western blot-positive samples possessing IgG or IgM antibodies than the use of CDC criteria (Χ^2^ = 6.9, *p* < 0.01). In patients negative in the LM-PCR assay, the positivity rates for IgG and IgM antibodies in western blots were the same with both criteria. Although the western blot positivity rate for any antibody was higher by the IGeneX criteria than by the CDC criteria when the results from all 107 patient samples were considered together, the difference was not statistically significant (Χ^2^ = 2.3, *p* > 0.05). Twenty-three patients (21.5% of the cohort) who tested negative for either IgG or IgM antibodies to BB by the CDC criteria, and 12 (11.2% of the cohort) by the IGeneX criteria, were identified to have BB-specific DNA by the LM-PCR assay.

The numbers of patients with BB infections detected by the LM-PCR assay, but who did not possess detectable IgG ant-BB antibodies by the CDC and IGenex criteria were 30 and 25, respectively, corresponding to 28.0% and 23.4% of this cohort. Fourteen patients or 13.1% of the Texas cohort were negative in the LM-PCR assay but positive for IgG antibodies to BB by both CDC and IGeneX criteria.

### Results of LM-PCR and western blot assays on IGeneX clinical samples

Only 402 of 5,964 samples were positive by the LM-PCR assay performed on whole blood and serum over a period of 2 years (representing 6.7% of the 5,964 samples). The results of western blots performed on the 402 samples and judged as positive by the CDC and IGenex criteria are shown in Table 3.

**Table 3:** Analysis of western blot results from the 402 LM-PCR assay-positive IGeneX samples

Western blot result	Total antibody positive	Isotype of anti-BB antibody response detected		
		Only IgM	Only IgG	Both IgG and IgM
Positive by IGeneX criteria	236	147	57	32
Positive by CDC criteria	70	60	7	3

The difference between proportions detected using IGeneX and CDC criteria is significant for every column at the *p* < 0.0001 level by the chi-square test

As shown in the table a significantly higher proportion of LM-PCR positive patient samples were positive for IgM and IgG antibodies to BB by WB with the IGeneX criteria as compared to CDC recommended criteria (*p* < 0.0001).

If only IgG antibodies to BB are considered as relevant for serological identification, of all the patients tested at IGeneX over the 2 years, 392 (6.6%) using the CDC criteria and 313 (5.2%) using the IGeneX criteria could be identified as having BB infection only through the LM-PCR assay.

## Discussion

The results with clinical samples demonstrate that PCR can be performed on blood, serum and urine samples using a hybrid-select Lyme multiplex PCR-dot blot procedure termed the LM-PCR assay. The assay uses magnetic beads coated with streptavidin to select for BB DNA hybridized to the biotin-labeled BB-specific capture probes. The selected BB DNA fragments bound to the magnetic beads are washed several times to remove non-BB DNA, potential PCR inhibitors and other cell debris. The hybridization selection method using BB-specific capture probes was designed to be more efficient in specifically extracting BB DNA than the Gentra column procedure. It is well documented that inhibitors that interfere with PCR are found in clinical samples such as blood and urine [15, 17]. Several other methods have been described for extracting sufficient quantities of clean DNA free of PCR-inhibitory substances from clinical specimens [17, 18]. However, these methods are lengthy, time-consuming and subject to loss of target DNA during the extraction procedure. Substances normally present in blood, serum and urine were shown not to inhibit PCR amplification after hybrid-selection of BB DNA in the LM-PCR assay. The high sensitivity of the LM-PCR assay is probably due to the removal of potential PCR inhibitors and the higher and more consistent yield of DNA in the hybrid-select procedure that in turn provided approximately 100-fold greater sensitivity with urine samples and 10-fold greater sensitivity with serum samples than the widely used Gentra column method for isolating DNA. Interestingly, the Gentra method was as sensitive as the hybrid-select procedure for whole blood samples. The reason for the difference with whole blood samples is not clear but may be related to the presence of other cells in blood. Greater sensitivity is particularly important for detection of BB, because the bacteria are typically found in very low concentrations in blood and urine samples from Lyme disease patients.

The LM-PCR assay is highly specific for BB because it did not detect any of other pathogens tested. The PCR primers showed no cross reaction with any of the control human samples, control pathogen cell lysates or DNA that were tested. Furthermore, DNA sequencing of the PCR products from patient samples that were positive in the LM-PCR assay showed that the amplified DNA was derived from BB. The potential drawback with PCR-based diagnosis in Lyme disease, particularly with urine samples, has been the carryover of amplified DNA between samples [26]. Several precautions were taken to avoid this problem in our procedure. In every run, positive and negative controls were included to monitor the sample processing step, PCR and cross-contamination. Specimen separation was performed in a different room than the PCR assay. Initial processing was performed in a class II biohazard cabinet dedicated to PCR sample processing. PCR preparation was performed within a dedicated PCR hood. All the PCR reagents were aliquoted within the reagent preparation hood. Processed clinical sample DNA, and PCR positive and negative controls were added to the PCR reaction mix in another dedicated PCR hood. Amplification occurred in thermocyclers located in a separate work area. Assay runs, where any of the positive controls were negative or any of the negative controls were positive due to contamination, were discarded and the samples retested.

Despite similar overall limits of detection of the plasmid encoded *OspA* gene and the genome encoded *flagellin* gene from cultured BB cell lysates, some variability in the relative detection was observed in individual clinical samples, as reported also by others [27]. Therefore multiplex detection of both genes in one test, as done in the LM-PCR assay, is preferable.

Antibodies of the IgM class are the first to be produced in an infection but tend to be more cross-reactive between infectious organisms. The presence of IgM antibodies is recommended to be disregarded for diagnosis of Lyme disease by the CDC in samples obtained >30 days after the onset of symptoms [5, 6]. The presence of IgM antibodies to BB in a high proportion of both the Texas and IGeneX samples may therefore not be conclusive proof of BB infection since the majority, if not all, samples are likely to have been derived from persons who had been infected with BB for more than 30 days in view of the characteristics of the test referral process in both cohorts. However, the formation or persistence of IgM anti-BB antibodies in the later chronic-infection stage of Lyme disease has been documented [28–31]. Further exploration of the possible diagnostic value of IgM anti-BB antibodies in chronic infection with BB may therefore be warranted.

The results show that using the in-house developed IGeneX criteria resulted in a higher proportion of western blot positive samples than the presently CDC-recommended criteria in patients shown to have BB infections by the LM-PCR assay. More extensive studies in applying the two criteria are needed to further examine their relative utility for diagnosing Lyme disease. The detection of BB DNA in the LM-PCR assay is indicative of an ongoing infection or perhaps a very recently cured infection with BB. The LM-PCR assay differs in this sense from western blot serological assays because antibodies to a bacterial pathogen persist for long periods after the pathogen is eliminated, while pathogen DNA is very rapidly lost from the body.

In the present study, 28% of the entire Texas cohort and 6.6% of a much larger IGeneX sample population were positive only by the LM-PCR assay, and not by IgG western blots according to the CDC criteria. Such patients have gone undiagnosed if only the presently recommended diagnostic western blots criteria had been strictly employed. However, it is also clear from the present data that serological detection of BB infection using western blots is essential in many cases. BB is known to use different mechanisms to subvert a protective host immune response, including locating itself to relatively immunologically inaccessible tissues (e.g. the central nervous system) and actively interfering with host immune mechanisms [32, 33]. BB is also capable of enclosing itself in protective biofilms in extracellular matrices [34] which further reduces exposure to the host immune system. Exploitation of various immune evasion mechanisms by BB in chronic Lyme disease, as well established for diseases like malaria that often produce chronic infections in humans [35], can result in poor serum antibody responses but yet a DNA signature of BB infection may be picked up in a sensitive and specific PCR. To our knowledge, all patients positive only in the LM-PCR assay were subsequently treated for Lyme disease by the referring physicians. Further studies on the clinical characteristics and outcome of treating patients showing only a positive LM-PCR assay result may be valuable.

Location of BB in the urinary bladder is also known to occur and detection of Osp A protein in urine is reported to be a sensitive diagnostic procedure [36]. The shedding of BB bacteria and/or plasmids containing the genes for OspA and flagellin from the bladder may be responsible for their detection in the urine of the majority of Texas patients who were positive in the LM-PCR assay. The results suggest that testing urine samples by the LM-PCR assay is important for patients suspected to have Lyme disease.

In summary, the findings suggest that (1) the currently recommended CDC criteria for positivity in IgM and IgG western blot assays [5, 6] may need to be optimized, and (2) a PCR-based test such as the LM-PCR assay described here, applied to blood and urine samples, can be an important and perhaps necessary supplement to the presently recommended two-tier serological tests [5, 6] for more effective laboratory diagnosis of patients with Lyme disease.
